# Periodontal Health Among Pregnant Women in Sri Lanka: A Cross‐Sectional Study

**DOI:** 10.1002/puh2.209

**Published:** 2024-07-03

**Authors:** Kavithrini Anunadika Gammulle, Manori Dhanapriyanka, Meghashyam Bhat

**Affiliations:** ^1^ Ministry of Health Colombo Sri Lanka; ^2^ School of Dentistry University of Queensland, Australia Brisbane Australia; ^3^ School of Dentistry The University of Adelaide Adelaide Australia

**Keywords:** gingivitis, health services accessibility, oral hygiene, periodontal diseases, pregnant women, socioeconomic factors, Sri Lanka

## Abstract

**Introduction:**

The prevalence of periodontal disease among Sri Lankan women in the reproductive age group is considerably high. The present study aimed to assess the oral hygiene status, gingival status, and the associated factors among pregnant women attending community Antenatal Clinics (ANC) in Sri Lanka, against the challenging sociopolitical backdrop.

**Methods:**

This cross‐sectional study was conducted among 576 pregnant women attending community ANCs within a specific Medical Officer of Health area in Sri Lanka. The clinics were selected using a two‐stage cluster sampling method with probability proportionate to size technique. Pregnant women were recruited from each clinic using a systematic sampling method. Data was collected with an interviewer‐administered questionnaire and an oral health examination form.

**Results:**

Poor oral hygiene was detected among over 60% of pregnant women. Moderate‐to‐severe gingivitis was seen among 23.3% of them. Nearly 67% of the participants demonstrated limited knowledge of periodontal diseases. Approximately, 67% of them were unemployed, and 32.5% had a monthly income of less than 40,000 Sri Lankan rupees. Regression analysis revealed that the trimester, socioeconomic factors, frequency of dental visits, recency of full mouth scaling, and knowledge of periodontal diseases predicted the oral hygiene status, and age, trimester, frequency of brushing, recency of full mouth scaling, and knowledge of periodontal diseases were significant predictors of gingival health. Particularly, individuals with a lower socioeconomic status experienced both poor oral hygiene and gingival health.

**Conclusion:**

The suboptimal oral hygiene and gingival health, limited access to dental care, and inadequate periodontal health knowledge, highlight an urgent need for interventions such as empowering young women through educational and employment initiatives.

## Graphical abstract

The findings of the present study involving 576 pregnant women in Sri Lanka reveal alarming knowledge gaps related to periodontal health, coupled with a higher incidence of poor oral hygiene and gingivitis. The study emphasizes the significance of regular dental visits during pregnancy and underscores the importance of focusing on socioeconomic disparities to enhance oral health. It recommends targeted interventions aimed at empowering women.

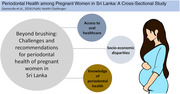

## 1 Introduction

Periodontal diseases include a wide range of inflammatory conditions that affect the supporting structures of the teeth which could lead to tooth loss and systemic inflammation [1]. According to the Global Burden of Disease Study 2019, there were 1.1 billion prevalent cases of severe periodontitis globally [2]. Risk factors for periodontal disease include lifestyle factors such as smoking, alcohol consumption, poor oral hygiene, systemic diseases such as diabetes mellitus, obesity, metabolic syndrome, osteoporosis, and genetic factors [3].

Pregnant women, being more susceptible, exhibit a high prevalence of gingival problems and periodontal diseases, estimated at around 40% [4, 5]_._ The scientific evidence for the association between periodontal disease in pregnancy and adverse pregnancy outcomes cannot be ignored [6]. A study among pregnant women in Croatia found inadequate awareness regarding the significance of maintaining good oral hygiene during pregnancy [7].

A study conducted in the western province of Sri Lanka in 2012 [8] reported a staggering 93% prevalence of periodontal disease among pregnant women, whereas National Oral Health Surveys conducted in the country indicated an improvement in the periodontal status of women in the reproductive age group over the past three decades [9].

The most recent investigations into the periodontal health status of pregnant women in Sri Lanka occurred over a decade ago, involving smaller sample sizes and lacking examination into the association between periodontal health and factors such as knowledge about periodontal diseases, socioeconomic variables, and access to dental care [8, 10].

Sri Lanka's economic setback and increasing poverty rates [11] significantly affect the vulnerable groups in society including pregnant women [12]. Providing health care to them has become a public health challenge due to the depletion of health resources, transportation, and financial barriers [13]. However, the guidelines for the provision of oral health care to pregnant women follow a universal policy applicable to pregnant women of all social strata despite the current challenging socioeconomic environment.

The insights provided by this study will be particularly important for policymakers to identify the need to address the varying levels of oral health requirements of pregnant women from different socioeconomic strata. The study's substantial sample size and its focus on factors such as knowledge, socioeconomic variables, and access to dental care and their association with periodontal health bring novelty to it. This research aimed to comprehensively assess the oral hygiene and gingival status of pregnant women attending community Antenatal Clinics (ANCs) in Sri Lanka and identify their association with knowledge of periodontal diseases, socioeconomic status, and access to dental care services.

## 2 Methods

### 2.1 Study Design, Setting, Sample Size, and Sampling Method

This cross‐sectional study was conducted from August 2019 to December 2019, among pregnant women in the second and third trimesters, who attended ANCs in the Piliyandala Medical Officer of Health (MOH) area, Sri Lanka. Those with diabetes mellitus, endocrine disorders, risk of developing infective endocarditis, and taking calcium channel blockers, antiepileptic medications, or other drugs known to cause gingival hyperplasia were excluded.

The sample size was determined using the formula for estimating a population proportion with absolute precision [14]. Considering the prevalence of periodontal diseases among pregnant women in a study conducted in Nepal (40%) [15], at a 95% confidence level and accepting a sampling error of 5%, a minimum sample of 369 was required. Further, making an allowance for a 1.5% design effect for cluster sampling and a nonresponse rate of 5%, the final sample size required was 581.

The sample was selected through a two‐stage sampling approach. The number of pregnant women to be selected from each ANC was based on the probability proportionate to the size method, based on the annual attendance figures for 2018 at each ANC. A systematic sampling technique was employed to select the designated number of participants from each clinic. The first participant was chosen randomly using the random number generator. Then every other participant was selected based on the order they were sitting until the desired sample size was achieved.

### 2.2 Data Collection Method

The pretested, interviewer‐administered questionnaire included items on sociodemographic factors, tooth brushing practices, access to dental care, and knowledge about periodontal diseases. It was validated by two experts in periodontology and dental public health, followed by a pilot study conducted among 20 pregnant women in the Boralesgamuwa MOH area. Subsequent revisions were made to enhance the face and content validity of the questionnaire. Knowledge about periodontal diseases was assessed using eight multiple‐choice questions on the effects of poor oral hygiene on periodontal health, prevention of periodontal diseases, risk factors for periodontal diseases, and recurrence of periodontal diseases following treatment. Those who obtained a score of 50% or more for the items on knowledge were categorized as having good knowledge, whereas those who scored less than 50% were categorized as having poor knowledge.

Periodontal health was assessed by examining the oral hygiene and gingival status. The Oral Hygiene Index Simplified (OHI‐S) of Greene & Vermillion [16] and the Gingival Index (GI) of Loe & Silness [17] were used to assess the oral hygiene status and the Gingival status, respectively. The first author conducted the oral examinations following calibration against a professor in periodontology. The Kappa values for interexaminer variability for OHI and GI were 0.7 and 0.8, respectively.

Oral hygiene status was categorized as good (OHI‐S score = 0.0–1.2), satisfactory (OHI‐S score = 1.3–3.0), and poor (OHI‐S score = 3.0–6.0) [18]. Gingival inflammation was categorized as “0”—no inflammation, “1”—mild inflammation with slight erythema or edema, but no bleeding on probing, “2”—moderate inflammation with moderate erythema and edema and bleeding‐on‐probing, and “3”—severe inflammation with marked erythema and hypertrophy and spontaneous bleeding [19]. Questionnaires were administered to each participant. Following this, the oral examination was conducted within the ANC premises using sterile instruments, while the participant was seated on a straight‐back chair. After data collection, the pregnant women who required dental care were referred to the nearest dental clinic.

### 2.3 Data Analysis

Data analysis was carried out using IBM Statistical Package for Social Sciences [SPSS] software version 21. GI and OHI—S were the dependent variables, whereas the independent variables consisted of knowledge, socioeconomic factors, and access to dental care. Univariate analysis was carried out for each independent variable using an independent sample *t*‐test. Linear regression was initially utilized to assess the association between knowledge, oral hygiene status, and gingival status. Residuals of multiple linear regression models for both the OHI‐S and GI scores were normally distributed, VIF values were less than 2.00, and homoscedasticity of the residuals was observed. Hence, multiple linear regression analysis with backward stepwise elimination was used to evaluate the impact of all independent variables on oral hygiene and gingival status. The regression model initially included the variables selected by the univariate analysis and those associated with the two dependent variables (OHI and GI) according to the literature.

## 3 Ethics Considerations

Ethical approval was obtained from the Ethics Review Committee, Postgraduate Institute of Medicine, University of Colombo (ERC/PGIM/2019/105). All procedures performed in the study were per the ethical standards of the institute and with the 1964 Helsinki Declaration and its later amendments.

## 4 Statement of Informed Consent

Every participant was provided with information on the study, and informed written consent was obtained for their anonymized information to be disseminated following the research.

## 5 Results

The response rate was 99.1%. The sample was predominantly Sinhalese with a percentage of 80.2. The average age of the participants was 29.2 years (SD ± 4.8 years) and 66.5% were less than 30 years old. Many participants (58.3%) were in the second trimester (Table 1).

**TABLE 1:** Demographic profile, socioeconomic characteristics, and access to dental care of pregnant women attending community antenatal clinics, Piliyandala, Sri Lanka, 2019.

Characteristics	Study participants	
	Number	Percentage
**Age group—years**		
16–30	383	66.49
31–35	193	33.51
**Trimester**		
Second trimester	336	58.37
Third trimester	240	41.63
**Years of education**		
<13 years	484	84.03
>13 years	92	15.97
**Current status of employment**		
Employed	192	33.33
Unemployed	384	66.67
**Average monthly household income Rs**.		
<40,000	187	32.47
>40,000	389	67.53
**Last visit to a dentist**		
Within the last 12 months	277	48.1
More than 1 year ago	289	51.9
**Treatment obtained on the last visit**		
Scaling	49	8.5
Other	527	91.5

### 5.1 Knowledge About Periodontal Disease

**FIGURE 1:**
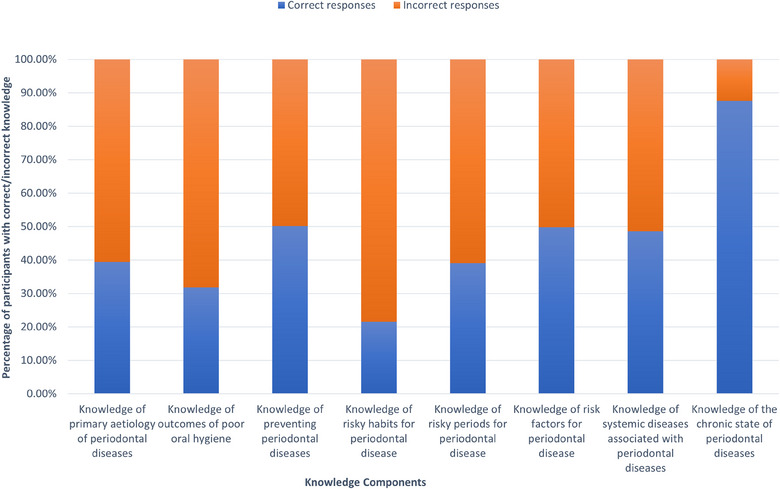
Knowledge of periodontal diseases among pregnant women Attending Community Antenatal Clinics, Piliyandala, Sri Lanka, 2019..

Nearly 67% had poor knowledge about periodontal diseases. Around 57% believed that periodontal diseases resulted from collection of food particles. Only about 13% knew that pregnancy is a high‐risk period for periodontal diseases (Figure 1).

The means of OHI‐S and GI scores were significantly different when the two knowledge groups were compared (Table 2) and knowledge statistically significantly predicted oral hygiene status and gingival status (Table 3).

**TABLE 2:** Univariate analysis: Comparison of means of Oral Hygiene Index Simplified (OHI‐S) and Gingival Index (GI) of pregnant omen Attending Community Antenatal Clinics, Piliyandala, Sri Lanka, 2019.

	OHI‐S		GI	
Variable	Mean	SD	Mean	SD
**Age group‐years**				
16–30	1.54	1.03	**0.45**	**0.43**
31–45	1.68	0.89	**0.87**	**0.46**
**Stage of pregnancy**				
Second trimester	1.6	0.89	**0.75**	**0.43**
Third trimester	1.7	0.95	**0.82**	**0.46**
**Years of education**				
<13 years	1.69	1.03	**0.84**	**0.48**
>13 years	1.59	0.79	**0.73**	**0.40**
**Employment status**				
Employed	1.55	0.89	0.77	0.43
Unemployed	1.69	0.93	0.79	0.46
**Monthly income**				
<40,000	1.70	0.90	**0.81**	**0.46**
≥40,000	0.57	0.92	**0.74**	**0.42**
**Type of dentifrice used**				
Fluoridated toothpaste	1.65	0.91	**0.87**	**0.48**
Herbal toothpaste	1.63	0.93	**0.76**	**0.43**
**Frequency of brushing**				
Once a day	1.78	0.91	0.90	0.54
Twice or more	1.63	0.92	0.77	0.46
**Last visit to a dentist**				
>1 year	**1.74**	**0.96**	0.78	0.45
≤1 year	**1.51**	**0.83**	0.77	0.43
**Full mouth scaling**				
Received within a year	1.50	0.92	**0.69**	**0.42**
Not received	1.68	0.91	**0.80**	**0.45**
**Knowledge**				
Poor	1.82	0.95	**0.86**	**0.48**
Good	1.29	0.73	**0.63**	**0.29**

*Note*: Bold signifies that the means are significantly different at the *p* < 0.05 level.

**TABLE 3:** Simple regression analysis: impact of knowledge on Oral Hygiene Index Simplified (OHI‐S) and Gingival Index (GI) among pregnant women attending community antenatal clinics, Piliyandala, Sri Lanka, 2019.

	Oral Hygiene Index‐S				Gingival Index			
	95% CI for β				95% CI for β			
Variable	Unstandardized coefficient (*β*)	Lower limit	Upper limit	Significance	Unstandardized coefficient (*β*)	Lower limit	Upper limit	Significance
Knowledge	−0.53	−0.68	−0.38	<0.05	−0.23	−0.31	−0.16	<0.05

Abbreviation: CI, Confidence interval.

### 5.2 Oral Hygiene Status

The mean OHI‐S score of the study population was 2.39 ± 1.35. The mean OHI‐S scores of those in the second and third trimesters were 2.37 and 2.41, respectively. In that order, Good, satisfactory, and poor oral hygiene were observed among 9.2%, 29.2%, and 61.6%.

Univariate analysis depicted statistically significant differences in OHI‐S scores between subcategories of the last dental visit and level of knowledge (Table 2). Backward stepwise regression analysis revealed that being in the third trimester, having less than 13 years of education, being unemployed, having a low income, not having a dental visit within a year, not receiving full mouth scaling within a year, and having good knowledge on periodontal diseases were all statistically significantly associated with oral hygiene status (Table 4).

**TABLE 4:** Stepwise backward regression analysis: predictors of Oral Hygiene Index Simplified (OHI‐S) among pregnant women attending community antenatal clinics, Piliyandala, Sri Lanka, 2019.

	Oral Hygiene Index‐S			
		95% CI for *β*		
Variable	Unstandardized coefficient (*β*)	Lower limit	Upper limit	Significance
Third trimester	0.13	−0.02	0.27	0.09
<13 years of education	0.30	0.15	0.45	0.00
Unemployment	0.28	0.12	0.44	0.00
Low income	0.15	0.01	0.29	0.04
Last dental visit >1 year ago	0.29	0.14	0.44	0.00
Not receiving full mouth scaling within a year	0.21	0.03	0.39	0.02
Good knowledge	−0.59	−0.74	−0.43	0.00

Abbreviation: CI, Confidence interval.

### 5.3 Gingival Status

The mean GI value was 0.78 (SD ± 0.44). Similar to the oral hygiene status, the mean GI score was higher in the third trimester (0.82) compared to the second (0.75). Moderate‐to‐severe gingivitis was observed in 23.3% of the participants.

Univariate analysis revealed that the differences in mean GI scores according to age, trimester of pregnancy, years of education, status of employment, monthly income, type of dentifrice, full mouth scaling, and knowledge were statistically significant.

According to stepwise backward regression, being less than 30 years of age, being in the third trimester, brushing teeth less than twice a day, not receiving full mouth scaling within a year, and having good knowledge about periodontal diseases predicated GI statistically significantly (Table 5).

**TABLE 5:** Stepwise backward regression analysis: predictors of Gingival Index (GI) among pregnant women attending community antenatal clinics, Piliyandala, Sri Lanka, 2019.

	Gingival Index			
		95% CI for *β*		
Variable	Unstandardized coefficient (*β*)	Lower limit	Upper limit	Significance
Age <30 years	−0.09	−0.17	−0.02	0.02
Third trimester	0.09	0.02	0.16	0.01
Brushing frequency less than twice a day	0.15	0.00	0.30	0.04
Not receiving full mouth scaling within a year	0.11	0.02	0.19	0.01
Good knowledge	−0.23	−0.31	−0.16	0.00

Abbreviation: CI, Confidence interval.

## 6 Discussion

This study investigated the periodontal status of pregnant women in Sri Lanka. Although earlier studies have explored the impact of pregnancy on periodontal health, they have not explicitly specified the complex interaction between knowledge, socioeconomic status, access to dental care services, and the periodontal health of pregnant women.

The study participants were mostly Sinhalese under 30 years old, with many in their second trimester of pregnancy. Most participants had poor knowledge of periodontal disease, with significant associations found between knowledge levels and oral hygiene as well as gingival status. Factors such as trimester of pregnancy, education, employment, income, recent dental visits, receiving full mouth scaling, and knowledge about periodontal diseases were significant predictors of oral hygiene, whereas age, trimester of pregnancy, brushing frequency, recency of full mouth scaling, and knowledge about periodontal diseases were significant predictors of gingival status.

Our findings suggest that pregnant women may experience improved periodontal health through better awareness of periodontal diseases, regular dental visits, provisions from public sector interventions, and interventions via other relevant authorities such as private sector, and nongovernmental organizations aimed at enhancing their socioeconomic conditions.

Although the current study highlights the critical need for adequate knowledge about periodontal diseases for better periodontal health, previous regional studies have similarly shown a lack of knowledge among pregnant women [20]. The current participants believed the accumulation of food particles caused periodontal diseases and were not aware that they were at a higher risk for poor periodontal health during pregnancy.

The majority of the pregnant women in our study experienced poor oral hygiene. Those in the third trimester had poorer oral hygiene and gingival status than the others. However, few follow‐up studies [8, 16] found that the periodontal status deteriorated with the progression of the trimesters.

Our study suggests that targeted educational campaigns integrated into prenatal care programs are required to bridge the existing knowledge gap effectively. In the future, longitudinal studies can be planned to assess the effectiveness of such interventions and compare the outcomes of different educational strategies. Future studies may also explore the impact of programs that address the socioeconomic disparities among pregnant women on their periodontal health. The need for qualitative studies to assess the barriers and facilitators to accessing dental care services among pregnant women can also be highlighted here.

Nevertheless, assessing self‐reported data such as oral hygiene practices can introduce bias to the study, and longitudinal studies are warranted to understand the temporal interplay among socioeconomic factors, knowledge levels, access to dental care, and periodontal health. Furthermore, the study population was predominantly Sinhalese as well as it does not encompass pregnant women who do not attend the government‐sponsored community antenatal clinics. Therefore, the results have potential limitations in generalizability.

Overall, oral hygiene and gingival health were suboptimal in this cohort of pregnant women. Limited access to dental care, poor socioeconomic status, and inadequate knowledge about periodontal health exacerbate the current situation. These findings offer policymakers invaluable insights for tailoring equitable service provision to meet the periodontal health needs of pregnant mothers during this challenging period.

## 7 Conclusion

The identified suboptimal oral hygiene and gingival health, along with limited dental care access and inadequate periodontal health knowledge, underscore the urgent need for interventions, such as empowering young women through educational and employment initiatives, given the high unemployment rates and the strong association between low socioeconomic status and adverse periodontal health outcomes. This study not only highlights an unacceptable knowledge gap regarding periodontal health and the importance of regular dental visits during pregnancy but also stresses the need to address socioeconomic inequalities to improve oral health, providing valuable insights for policymakers aiming for equitable and effective service provision in the current sociopolitical climate.

## Author Contributions

K.A.G was involved in the conception, data collection, and analysis, design of this study, and drafting of the manuscript. M.D drafted the initial manuscript and reviewed. M.B was involved in interpreting the data, critically reviewing the paper, and approving the final manuscript.

## Conflicts of Interest

The authors have no conflicts of interest to declare. All coauthors have agreed with the contents of the manuscript and there is no financial interest. We certify that the submission is original work and is not under review at any other publication.

## Data Availability

The data that support the findings of this study are available from the corresponding author upon reasonable request.
